# Prediction of Potential Distribution and Response of *Changium smyrnioides* to Climate Change Based on Optimized MaxEnt Model

**DOI:** 10.3390/plants14050743

**Published:** 2025-02-28

**Authors:** Xingyu Zhu, Xin Jiang, Ying Chen, Congcong Li, Shi Ding, Xuejiao Zhang, Lulu Luo, Yun Jia, Gang Zhao

**Affiliations:** 1 School of Medicine, Northwest University, Xi’an 710069, China; zhuxingyustudy@126.com; 2 College of Life Sciences, Northwest University, Xi’an 710069, China; 202133347@stumail.nwu.edu.cn (X.J.); 17392215506@163.com (Y.C.); lcc173351528432022@126.com (C.L.); shiding0621@163.com (S.D.); 18893043413@163.com (X.Z.); 18602922791@163.com (L.L.); 3 Xi’an Botanical Garden of Shaanxi Province (Institute of Botany of Shaanxi Province), Xi’an 710061, China

**Keywords:** species distribution model, environmental factors, migratory routes, *Changium* species, potential geographical distribution

## Abstract

*Changium smyrnioides*, an endangered herb known for its medicinal roots, contains essential amino acids that are vital for human health but cannot be synthesized by the body. However, wild populations of this species have been steadily declining due to the combined impacts of climate change and anthropogenic activities. In this study, we employed an optimized MaxEnt model to predict the potential distribution of *C. smyrnioides* under different climate scenarios and to evaluate its responses to climate change. Our findings demonstrated that the MaxEnt model achieved optimal performance with a regularization multiplier of 0.5 and a feature combination of linear and quadratic terms. Among the environmental variables, three emerged as the most critical factors shaping the species’ potential distribution: elevation, precipitation of the driest month (bio14), and isothermality (bio2/bio7 × 100, bio3). Currently, the primary suitable habitats for *C. smyrnioides* are concentrated in Jiangsu Province, with an estimated 21,135 km² classified as highly suitable. The analysis further indicated that, in response to rising temperatures, *C. smyrnioides* is likely to shift its distribution northeastward across China. Notably, the results suggested that the total area of suitable habitats would increase over time under projected climate scenarios. Based on the predicted centroid migration of suitable habitats, Anhui Province was identified as a critical future conservation zone for *C. smyrnioides*. This region could serve as a vital refuge, ensuring the long-term survival of the species under changing climatic conditions. Overall, this study provides key insights into the ecological responses of *C. smyrnioides* to climate change, offering evidence-based guidance for the development of effective conservation strategies aimed at safeguarding this endangered herb.

## 1. Introduction

*Changium smyrnioides* (Mingdangshen) is a well-known medicinal herb native to East China, belonging to the genus *Changium*. It is traditionally used for its therapeutic properties in clearing the lungs, resolving phlegm, calming the liver, harmonizing the stomach, detoxifying the body, and treating conditions such as cough, wheezing, dizziness, vomiting, red eyes, and leukorrhea caused by phlegm and heat [1]. The plant has a spindle-shaped or long-cordiform root, measuring 5–20 cm in length, with a brownish-yellow or pale yellow surface and a white interior [2]. In daily life, the root is often sliced and brewed as tea, and is known for being rich in amino acids that the human body cannot synthesize [2]. *Changium smyrnioides*, an endangered species endemic to China, has a limited distribution range. Overharvesting and anthropogenic disturbances have contributed to a steady decline in both its population and range, resulting in habitat fragmentation and loss [3]. Although the impact of climate change on the population size and distribution of *C. smyrnioides* has yet to be directly studied, it is widely recognized that climate change can contribute to the reduction in species populations and the alteration of their native habitats, which may potentially affect the distribution and viability of species [4,5,6]. In 1984, it was listed as a National Level III Protected Plant in China [3], and in 2020, it was upgraded to a National Level II Protected Plant. Furthermore, it is classified as a Vulnerable species in the Red List of Endangered Species.

The IPCC Special Report on Climate Change reveals that the global average temperature over land increased by 1.41 °C during the period 1999–2018 compared to 1881–1900 [7]. Over the past few decades, the global climate has undergone significant shifts primarily driven by human activities, leading to heightened levels of greenhouse gasses and global warming [8]. Consequently, understanding the impact of climate change on species responses has become an important area of research [9]. Climate change not only threatens biodiversity and population dynamics, but it also significantly alters the geographical distribution of species [10,11], posing substantial risks to their survival. Therefore, investigating how species respond to climate change will not only help predict their potential future distributions but also provide critical insights for developing effective strategies in germplasm resource management and species protection [12]. For instance, scholars have proposed various important strategies for the conservation of endangered species, including the establishment of protected areas and ex situ conservation efforts [13,14].

Species distribution models (SDMs), based on the concept of ecological niches, were used to predict the potential distribution of species in the past or the future by combining known geographical distribution points and environmental variables [15,16], which can provide insights into the species migration and dispersal patterns [15,16,17,18]. Although various species distribution models, such as the Random Forest Model (RF), the Generalized Linear Model (GLM), the Categorical Regression Tree Model (CTA), and so on, are available, the Maximum Entropy Model (MaxEnt) has been widely recognized for its robust predictive performance across a range of data conditions [19]. While MaxEnt performs well with large datasets, it is particularly notable for its ability to generate reliable predictions even when species occurrence data are limited, making it widely regarded as a more robust model for species distribution prediction [20,21,22]. Furthermore, unlike some approaches that may require complex parameter tuning, targeted R packages “ENMeval” and “Kuenm” were developed to optimize default settings to achieve better predictive results [23,24]. Now, the MaxEnt model has been widely applied in various fields, including invasion biology and conservation biology. For instance, researchers have utilized the MaxEnt model to predict the global spread and proliferation patterns of *Chelydra serpentina* (native to North America) [25]. Additionally, the model has been combined with phylogenetic analysis and plastid genomics to assess species identification and the spread potential of invasive populations, such as the *Opuntia humifusa* complex in China [26]. In conservation biology, MaxEnt has proven invaluable, particularly in studies of endangered plant species. Notable examples include *Bergenia scopulosa*, a rare and endangered medicinal plant endemic to Shaanxi Province [27], and *Actinidia chinensis*, a National Level II Protected Plant in China [14]. By predicting potential habitats and migratory routes, MaxEnt facilitates the development of targeted conservation strategies for these at-risk species.

Based on the distribution records of *C. smyrnioides*, its primary habitats are concentrated in Zhejiang and Jiangsu provinces. Due to its significant medicinal value, current research on *C. smyrnioides* predominantly focuses on the pharmacological components derived from its roots [28]. This includes the extraction and analysis of root chemical constituents, such as the investigation into the distribution patterns of furanocoumarins, as well as a limited number of studies examining the population genetics of *C. smyrnioides* [29,30]. In recent years, however, the impact of human activities has led to a decline in wild populations, and its distribution range has become increasingly fragmented. Despite these concerning trends, there is a notable lack of research on how *C. smyrnioides* populations are responding to climate change. To address this gap, the present study aims to assess the potential distribution of *C. smyrnioides* under future climate change scenarios, identify the dominant environmental factors influencing its suitable habitats, and explore how these populations may adapt to changing climatic conditions. Additionally, this study seeks to provide targeted conservation strategies to safeguard this valuable species.

## 2. Results

### 2.1. Optimized Model and Performance Evaluation

**Figure 1:**
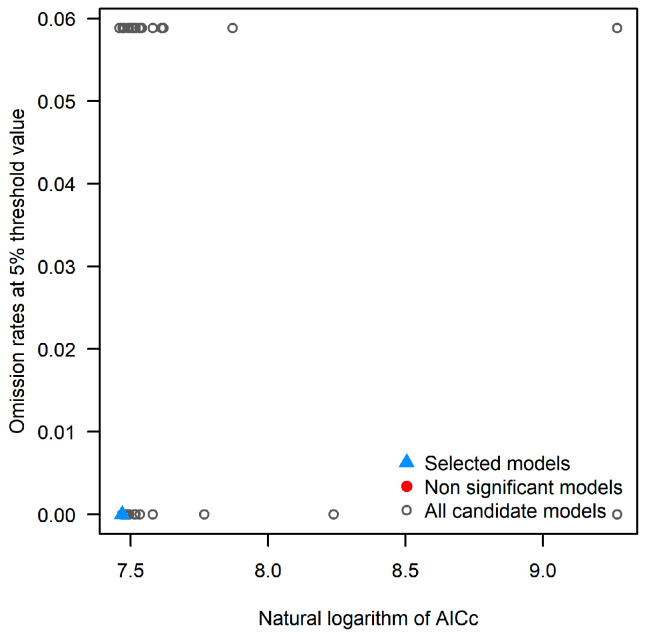
Omission rates and AICc values for all candidate models.

All models demonstrated statistical significance, with 25 candidate models meeting the omission rate criterion of ≤5%. Among these, only one model achieved the lowest AICc value, with an omission rate of 0.059 (Figure 1). Considering the three evaluation criteria—statistical significance, omission rate ≤ 5%, and delta AICc < 2—only one single model fully satisfied all the requirements to be designated as the optimal model. Therefore, the candidate model with an RM of 0.5 and an FC of “lq” exhibited the smallest delta AICc and was selected as the optimal configuration. A comparison between the default parameters and the optimized parameters revealed that the optimized model (RM = 0.5, FC = “lq”) had a significantly smaller delta AICc value than the default configuration (Table 1).

**Figure 2:**
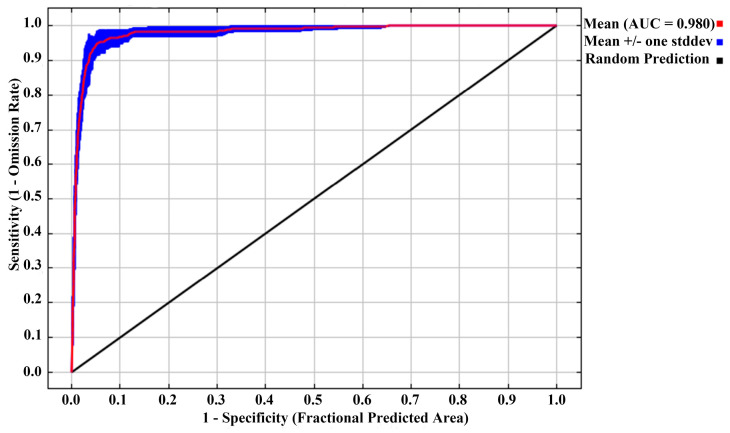
Evaluation of model performance: ROC curve of potential distribution prediction.

Furthermore, the performance of the MaxEnt model was assessed using the Receiver Operating Characteristic (ROC) curve. The analysis yielded an Area Under the Curve (AUC) value of 0.980, which significantly exceeded the threshold of 0.8, demonstrating the high predictive accuracy of the MaxEnt model in estimating the potential distribution of *C. smyrnioides* (Figure 2).

### 2.2. Analysis of Key Environmental Variables and Response Curve

**Figure 3:**
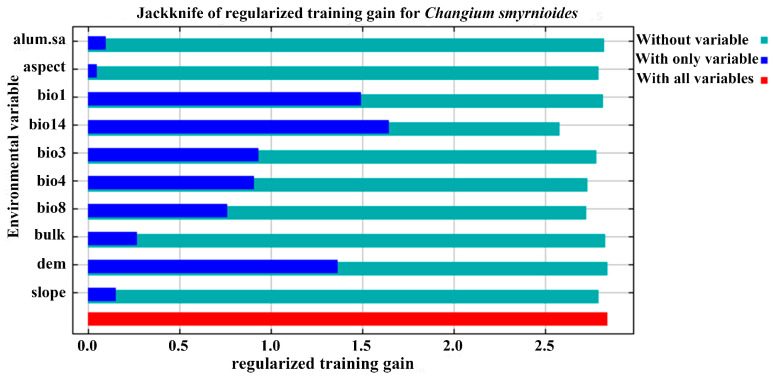
Jackknife test for environmental variables.

Based on the combined results of correlation analysis and variable contributions from the MaxEnt model, a total of 10 environmental variables were selected for further modeling (Supplementary Table S1). Among these, the dominant variables were elevation, precipitation of the driest month (bio14), isothermality (bio2/bio7 × 100, bio3), and temperature seasonality (bio4), which collectively accounted for more than 80% of the total contribution in determining the distribution of *C. smyrnioides* (Supplementary Table S2). In addition, a jackknife test was conducted to assess the significance of environmental variables in contributing to the regularized training gain. The results revealed that bio14 (precipitation of the driest month), bio1 (annual mean temperature), and elevation ranked as the top three variables. This highlights the critical role of bio14 and elevation in shaping the potential distribution of *C. smyrnioides* and their strong influence on the model’s regularized training gain (Figure 3).

**Figure 4:**
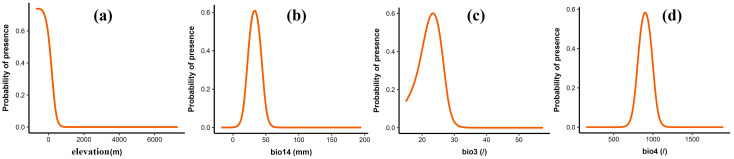
Response curves of four environmental variables in MaxEnt model for *C. smyrnioides*. (**a**) elevation; (**b**) bio14 (precipitation of the driest month); (**c**) bio3 (isothermality, bio2/bio7 × 100); (**d**) bio4 (temperature seasonality).

To evaluate the relationship between individual environmental variables and the probability of presence, logistic response curves were generated in MaxEnt and visualized using ggplot2. Environmental conditions were considered suitable for the growth of *C. smyrnioides* when the probability of presence exceeded 0.3. The corresponding suitable ranges for the key variables were as follows: elevation < 191 m, precipitation of the driest month (21.38 mm–44.74 mm), isothermality (19.31–26.87%), and temperature seasonality (8.0–10.02%) (Figure 4).

### 2.3. Modern Suitable Habitats for C. smyrnioides

**Figure 5:**
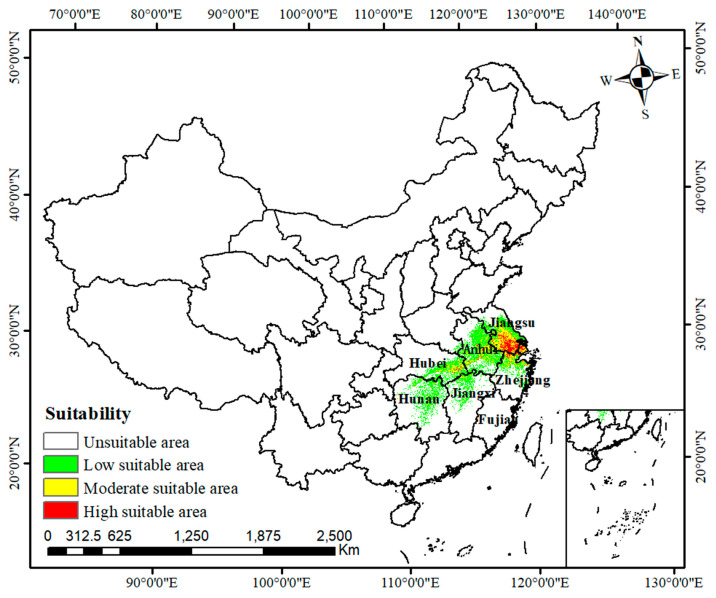
Prediction of potential suitable distribution of *C. smyrnioides* under current climatic scenarios.

The current potential distribution of *C. smyrnioides* is shown in Figure 5. Suitable habitats were primarily located in Jiangsu, Anhui, Zhejiang, Hubei, Hunan, and Jiangxi Provinces (Figure 5). Among these, highly suitable areas were concentrated in Jiangsu Province, covering 21,135.6 km^2^ (Table 2), which aligned closely with most of the species’ actual distribution records. Additionally, portions of moderately suitable habitats extended into Anhui, Hubei, and Zhejiang Provinces.

### 2.4. Prediction of Suitable Distribution in Future

**Figure 6:**
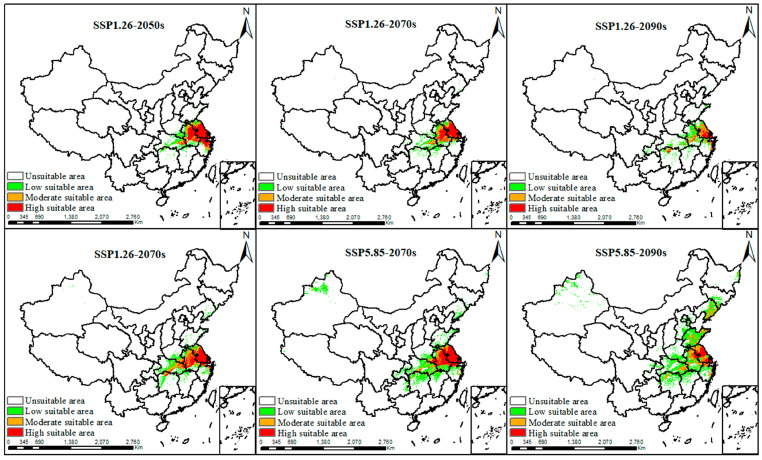
Potential suitable distribution of *C. smyrnioides* under future climatic scenarios.

To predict the potential distribution of suitable habitats in the future, we modeled the suitable habitats under two scenarios, SSP1.26 and SSP5.85, for the 2050s, 2070s, and 2090s, using MaxEnt v3.4.1(Figure 6). Under the SSP1.26 scenario, areas with high habitat suitability were projected to emerge in the Jiangsu, Anhui, and Zhejiang Provinces (Figure 6), with the 2050s showing the largest extent of high suitability as well as the total suitable areas (highly suitable area of 1.70 × 10^5^ km, and total suitable area of 3.78 × 10^5^ km^2^) (Table 2). Under the SSP5.85 scenario, high-suitability habitats in the 2050s were forecasted to occur primarily in Jiangsu, Anhui, and Hubei Provinces (Figure 6). By the 2070s, the high-suitability habitats would predominantly be concentrated in Jiangsu and Anhui, with Jiangsu still holding the largest area of high suitability (Figure 6). However, a part of the suitable habitats was expected to shift to Shandong and Liaoning under the SSP5.85 scenario in the 2090s (Figure 6). Notably, the total suitable areas for *C. smyrnioides* were projected to increase over time, primarily due to the expansion of low-suitability areas (Table 2).

### 2.5. Analysis of Dynamic Changes and Spatial Patterns

**Figure 7:**
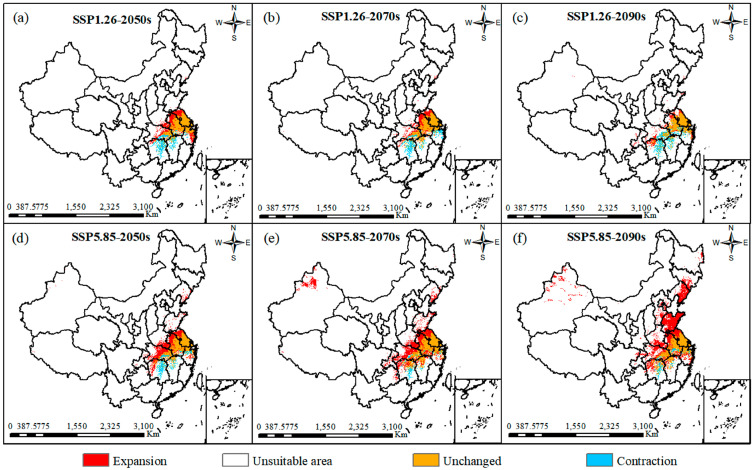
Spatial and geographical changes in the future under various climatic scenarios.

To investigate the response of *C. smyrnioides* to climate change under different scenarios, projections of the expansion, stable areas, and contraction of suitable habitats were generated by comparing future projections with the current distribution (Figure 7). The results showed that, under the SSP1.26 scenario, areas of habitat expansion predominantly occurred in northern Jiangsu, Anhui, southern Henan, and central Hubei Provinces. Under the SSP5.85 scenario, suitable habitats exhibited varying degrees of expansion, with the largest expansion occurring in the 2090s, covering an area of 6.039 × 10^5^ km^2^ (Table 3). In this scenario, the expansion was projected to further extend into Shandong, Hebei, Liaoning Provinces, and parts of the Xinjiang Uygur Autonomous Region. Overall, the suitable habitats of *C. smyrnioides* demonstrated a trend of northern expansion and southern contraction (Figure 7).

**Figure 8:**
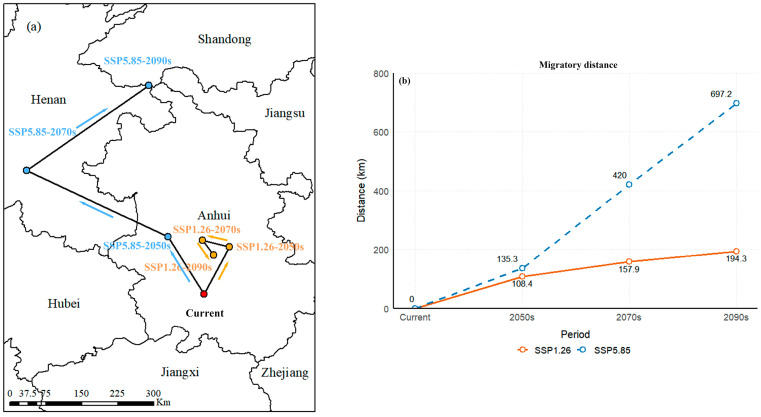
Migration of centroid of suitable habitats: (**a**) migratory routes; (**b**) migration distance. Arrows indicated migratory direction, while orange and green dots denoted centroids under the SSP126 and SSP585 scenarios, respectively.

To analyze the spatial patterns under future climate scenarios, we calculated the centroids of suitable habitats for each scenario and traced their migration routes (Figure 8). The results revealed a clear tendency for northward migration. Under the SSP1.26 scenario, the centroid would primarily remain in Anhui Province. In contrast, under the SSP5.85 scenario, the centroid would shift progressively from Anhui Province in the 2050s, to Henan Province in the 2070s, and finally to Shandong Province by the 2090s (Figure 8).

Compared to the relatively short migratory distance under the SSP1.26 scenario, the centroid under the SSP5.85 scenario would first move northwest within Anhui Province, covering a distance of 135.3 km. It would then continue its migration towards the northwest of Henan Province, at a larger angle, with a distance of 284.6 km, before settling in Henan. Finally, the centroid would shift northeast, covering 277.2 km, and settle in Shandong Province (Figure 8).

## 3. Discussion

### 3.1. Performance of Optimized MaxEnt Model

The MaxEnt model is widely used to infer species–environment relationships because of its ability to handle small sample sizes effectively and provide robust predictive performance [21,22]. This model has been applied across diverse fields, including the conservation of endangered species, assessment of climate change impacts on species distributions, and planning for ecological restoration [31,32,33,34]. Many researchers often adopt the default parameters of the MaxEnt model for simplicity [35,36,37]. However, several studies have pointed out that the default parameters may lead to increased model complexity and potential overfitting [38]. To improve model performance, we utilized the “Kuenm” package to assess candidate models by combining different values of the regularization multiplier (RM, ranging from 0 to 4 with a 0.5 interval) and feature class (FC) parameters, including l, lq, h, lqh, lqph, and lqpth. The results revealed that the optimized MaxEnt model, with RM = 0.5 and FC = lq, was the most effective, as it minimized both the omission rate and the delta AICc value (which was reduced to 0). The delta AICc value decreased from 2048.245 (using the default parameters) to 0 with the optimized model, indicating a significant reduction in complexity and overfitting [38]. Optimized parameters have been widely applied in previous studies to enhance the accuracy of MaxEnt predictions. For example, Shi found that for predicting *Magnolia wufengensis*, the optimal parameters were RM = 3.5 and FC = lq, which significantly reduced model overfitting [6]. Similarly, Wang demonstrated that with RM = 1.5 and FC = h, the model achieved a delta AICc of 0, and the 10% training omission rate was lower than when using the default parameters [39]. Additionally, Zheng identified that the best parameter combination for their model was RM = 4 and FC = qph, leading to highly accurate predictions [40].

### 3.2. Analysis of Key Environmental Variables in Geographic Distribution of C. smyrnioides

This study revealed that the dominant environmental variables in limiting the distribution of *C. smyrnioides* include elevation, precipitation of the driest month, and isothermality, among which factors bio14, elevation, and annual mean temperature (bio1) ranked as the top three in the jackknife test, further indicating the importance of the topographical variables elevation, precipitation, and temperature. This finding is supported by many similar studies. Zhao predicted the potential suitable habitats of *Saussurea* species, renowned for their biodiversity and medicinal significance in high-elevation regions, by using an optimized MaxEnt model and found that elevation, isothermality (bio3), and temperature annual range (bio7) were identified as the dominant environmental variables [31]. Fang revealed that the key environmental factors influencing the distribution of *Cirsium japonicum* were temperature annual range (bio1), precipitation of the driest month (bio14), and precipitation of the wettest month (bio13) [41].

Single-response curves have been widely used to unveil the impact of the range of environmental variables on the probability of species presence by many researchers [42,43]. Based on the range of the single-response curve of elevation, we found that the optimal range for the elevation variable was elevation < 191 m, when the presence probability exceeded 0.3. This was supported by the previous study of Wang, which found that *C. smyrnioides* was mainly located in eastern China, particularly at altitudes of 50–400 m above sea level [44,45], further validating the accuracy of the optimized MaxEnt model used in this study. High elevation often brings lower temperatures, increased humidity, and greater atmospheric pressure and radiation [46]. In such conditions, species are forced to expend more energy to adapt to the harsh environment, which can limit root growth and the synthesis of essential nutrients. This phenomenon can be explained by the “growth-defense trade-offs in plants” theory [47], which posits that energy allocated to defense mechanisms often comes at the expense of growth, especially in challenging environmental conditions. The range of precipitation during the driest month (bio14) was from 21.38 mm to 44.74 mm, with an optimal precipitation of 33.27 mm, suggesting that *C. smyrnioides* had relatively modest water requirements. A previous report documented that seed abortion was most severe during the dry season, primarily due to insufficient rainfall in 2000s [48], highlighting the importance of sufficient moisture for successful reproduction. Isothermality (Bio3) quantifies how large the day-to-night temperature fluctuations are relative to the summer-to-winter (annual) temperature oscillations [49,50]. An Isothermality value of 19–26.87% suggests that *C. smyrnioides* inhabits areas with relatively stable temperature conditions, where daily temperature fluctuations (diurnal range) are much smaller than seasonal temperature changes (annual range). These stable day-to-night temperature conditions are likely beneficial for species adapted to minimal daily temperature variation, but capable of tolerating more distinct seasonal temperature shifts. In contrast to alpine species that thrive in areas with large temperature fluctuations, *C. smyrnioides* prefers habitats with smaller diurnal temperature changes and is typically found at altitudes below 400 m [51]. Another key environmental variable, temperature seasonality (Bio4), has an optimal range of 8–10%, indicating moderate seasonal temperature variation. This suggests that *C. smyrnioides* likely inhabits regions where temperature fluctuates throughout the seasons, but without extreme variability. Such moderate temperature seasonality is associated with species having narrower thermal tolerances, which may explain their relatively lower elevational range compared to species adapted to more extreme seasonal temperature variations [52]. These findings suggest that *C. smyrnioides* thrives in environments with minimal temperature fluctuations; as such, stability likely promotes consistent metabolic and physiological processes, thereby enhancing its survival and growth in these regions. In contrast, greater temperature fluctuations may disrupt these processes, potentially causing irreversible damage to the plant’s metabolism and growth [53].

### 3.3. Potential Distribution of C. smyrnioides Under Climate Changes

**Figure 9:**
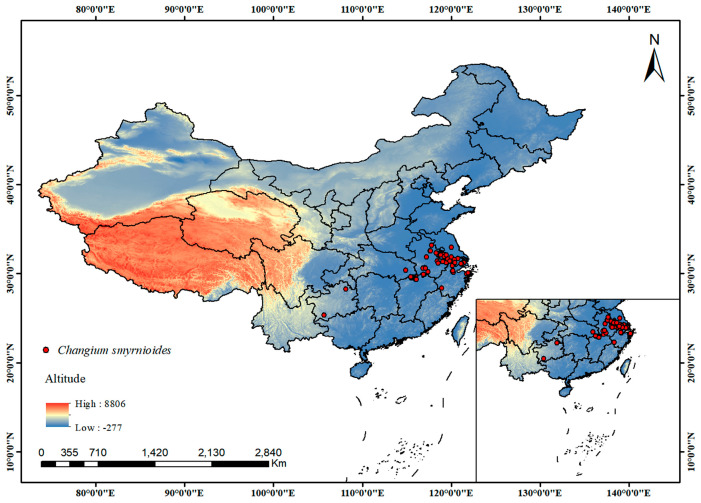
Geographic distribution of *C. smyrnioides* in China.

Based on the optimized MaxEnt model, this study provided a reliable framework to assess the potential impacts of future climate change on species distributions [54]. The current suitable habitats for the species were primarily concentrated in Jiangsu, Anhui, Zhejiang, Hubei, Hunan, and Jiangxi Provinces (Figure 9), closely corresponding to its recorded occurrences in Jiangsu, Zhejiang, and Anhui Provinces. This alignment demonstrated a high degree of reliability for the MaxEnt model. Similar findings have been reported for other species, including *Magnolia wufengensis*, Chinese *Ziziphus jujuba*, and *Primula filchnerae* [6,12,19].

Mounting evidence suggests that climate change can significantly alter species’ suitable habitats and global biodiversity [55,56]. For example, birds generally respond to climate change by either adapting to the new conditions without relocating or adjusting their geographical range to match the changing climate [55]. Plants similarly face pressures to adapt or migrate, leading to shifts in suitable habitats [57]. For instance, Zhang predicted that the suitable habitat of *Xanthium italicum* was likely to contract under future climate scenarios [58], while Zheng projected that *Cenococcum geophilum* would migrate to higher latitudes, increasing its suitable habitat from 9.21% to 21.02% [40]. These examples illustrated that climate change can exert contrasting effects on species, causing either habitat contraction or expansion. Under future climate scenarios (SSP1.26 and SSP5.85), both highly suitable and the total suitable areas for the species are projected to expand significantly. The highly suitable areas were expected to increase from 2.11 × 10⁴ km^2^ to peaks of 1.7 × 10^5^ km^2^ in the 2050s under SSP1.26 and 1.77 × 10^5^ km^2^ in the 2070s under SSP5.85. However, by the 2090s, these areas were predicted to decrease to 5.79 × 10^4^ km^2^ and 1.02 × 10^5^ km^2^ under SSP1.26 and SSP5.85, respectively. Similarly, the total suitable areas are predicted to expand from 2.96 × 10^5^ km^2^ to 3.78 × 10^5^ km^2^ under SSP1.26 in the 2050s, and then gradually decline to 3.03 × 10^5^ km^2^ by the 2090s. In contrast, under SSP5.85, total suitable areas were expected to exhibit a steady increase, reaching 8.75 × 10^5^ km^2^ by the 2090s (Table 2).

These changes highlight a general trend of expansion, particularly in the northern parts of the current suitable habitats, while contraction is predominantly observed in southern regions [12]. The net increase in suitable areas indicates that expansion outweighs contraction, consistent with observations in other temperature-sensitive plant species such as *Pseudomonas syringae*, *Rubus idaeus*, and *Platycodon grandiflorus* [59,60,61]. The migration patterns also differ between the two scenarios. Under SSP1.26, the migratory distance was shorter, approximately 194.3 km, compared to 697.2 km under SSP5.85 (Figure 8). As temperatures rise, the species tends to migrate longer distances, predominantly toward higher latitudes, aligning with studies showing that plants adapt to climate change by shifting their distributions to higher altitudes and latitudes [62]. Recent research emphasized that plant migration was largely influenced by biological traits in response to changes in temperature and precipitation [63,64]. This phenomenon underscores the broader pattern that temperature-sensitive species gradually migrate to higher altitudes and latitudes to adapt to the impacts of global warming [65].

### 3.4. Conservation Strategies

Given the endangered status of *C. smyrnioides* and its potential distribution under changing climate scenarios, urgent conservation measures are needed—not only to protect this species, but also to safeguard other threatened plants facing similar conditions [66]. It is widely recognized that biodiversity hotspots are key priorities for conservation efforts [67]. Many of the actual distribution records of *C. smyrnioides* aligned with areas of high biodiversity. However, a portion of its habitats was located near rural areas, which were particularly vulnerable to human activities.

To enhance the conservation of *C. smyrnioides* based on current and predicted conditions, we propose the following strategies:

(1) Establishment of targeted small-scale protected areas: these areas should focus on in situ conservation, as they provide suitable living conditions for *C. smyrnioides*, even under future climate change scenarios.
(2) Artificial cultivation in newly suitable areas: with the projected geographic expansion of suitable habitats under certain climate scenarios, provinces such as Liaoning and Shandong should be considered for the establishment of artificial cultivation areas to ensure species persistence.
(3) Germplasm conservation for contracted areas: in regions like Jiangxi and Hunan, where suitable habitats are expected to contract, the establishment of germplasm banks is essential. These banks can support genetic research into the species’ adaptive mechanisms in response to climate change and promote cultivation strategies.
(4) Strengthening legal frameworks and public awareness: comprehensive biodiversity protection laws and effective public outreach campaigns are critical for safeguarding *C. smyrnioides* and promoting sustainable conservation practices.

## 4. Materials and Methods

### 4.1. Occurrence Data Collection

*Changium smyrnioides* belongs to *Changium* and is a perennial herb. A total of 112 distribution records were collected from the Global Biodiversity Information Facility (GBIF, https://www.gbif.org/, accessed on 10 November 2024), the Chinese Virtual Herbarium (CVH, https://www.cvh.ac.cn/, accessed on 10 December 2024), the National Specimen Information Infrastructure (NSII, http://nsii.org.cn/2017/home.php, accessed on 10 December 2024), and the corresponding dissertation [68] (Figure 9, Supplementary Table S1). To minimize sampling bias and spatial autocorrelation, we used ENMtools v1.0 [69] to filter the distribution data. This process ensured that each grid cell retained only one occurrence point (1 km × 1 km), thereby improving the robustness of the dataset. After filtering, a total of 68 unique occurrence records were retained for further analysis (Supplementary Table S2).

### 4.2. Environmental Variables

We obtained 19 bioclimatic variables from the WorldClim global (version 2.1: https://worldclim.org/data/index.html, accessed on 5 October 2024) at a spatial resolution of 30 arc-seconds (~1 km) [70]. These variables included contemporary climate data for the period 1970–2000, as well as projections for three future periods: the 2050s (2040–2060), 2070s (2060–2080), and 2090s (2080–2100). Future projections were based on two Shared Socioeconomic Pathways (SSP1-2.6 and SSP5-8.5) using the BCC-CSM2-MR climate model (Beijing Climate Center Climate System Model) [71]. 

Topographic variables, including aspect and slope, were derived from ArcGIS software v 10.8, while elevation data were obtained from the Geospatial Data Cloud (https://www.gscloud.cn/#page1/2, accessed on 5 September 2024) [72]. Additionally, 11 soil variables—alum_sa, cec_cla, sand, silt, clay, total_n, ph_water, esp, tcarbon, cec_soil, and bulk density—were sourced from the Harmonized World Soil Database (HWSD, http://www.fao.org/faostat/en/#data, accessed on 6 September 2024) to account for soil properties in our analysis [73]. To ensure compatibility with the MaxEnt v3.4.1 modeling software [74], all environmental variables were processed to a uniform spatial resolution. In total, 33 environmental variables were included in the initial selection process for species distribution modeling (Supplementary Table S3). The variable selection process integrated the percentage contribution of variables derived from MaxEnt modeling and the results of correlation analysis performed using IBM SPSS Statistics V26.0. To ensure robustness and reduce multicollinearity, when the correlation coefficient between any two variables exceeded |0.8|, only one variable from each highly correlated pair was retained for subsequent analyses [75].

### 4.3. Establishment of MaxEnt Model and Performance Evaluation

To optimize the performance of the MaxEnt model, the R package “kuenm” [24] was used to evaluate combinations of Feature Class (FC) and Regularization Multiplier (RM) parameters. A total of 48 candidate models were generated by combining RM values ranging from 0.1 to 4.0 (in 0.1 intervals) with six FC configurations: “l” (linear), “lq” (linear and quadratic), “h” (hinge), “lqh” (linear, quadratic, and hinge), “lqph” (linear, quadratic, product, and hinge), and “lqpth” (linear, quadratic, product, threshold, and hinge) [40]. The default MaxEnt configuration (RM = 1, FC = lqph) was included as one of the candidate models. The 48 candidate models were evaluated and filtered using a three-step selection process: (1) statistically significant values, (2) omission rates below the criterion threshold of 5%, and (3) delta AICc values less than two [40,76]. The final model was selected based on the lowest delta AICc, balancing model complexity and goodness-of-fit [18]. Complexity and fit were assessed using the omission rate and Akaike Information Criterion corrected for small sample sizes (AICc) [24].

MaxEnt version 3.4.1 was used to predict the species’ potential habitats under various temporal and environmental scenarios. The dataset was divided into a 75% training set for model calibration and a 25% testing set for evaluation [77]. The background points were retained at the default settings, with the maximum number of background points set to 10,000. The MaxEnt model was repeatedly run 10 times, and the average training AUC value was automatically calculated [6]. To quantify the contribution of each environmental variable, a Jackknife test was conducted [78]. Response curves were generated to assess the effects of individual environmental variables on species presence probabilities. Model performance was evaluated using the area under the receiver operating characteristic Curve (AUC), which ranges from 0 to 1, with higher values indicating better predictive power [79]. Typically, an AUC value above 0.8 is considered indicative of strong model performance.

### 4.4. Classification of Suitable Habitats and Statistics

The outputs from the MaxEnt model were imported into ArcGIS 10.8 and converted into raster format for further reclassification of potential habitat suitability. Natural breaks were applied to categorize the habitats into four suitability classes: unsuitable habitats (*p* < 0.3), low-suitability habitats (0.3 < *p* < 0.5), moderately suitable habitats (0.5 < *p* < 0.7), and highly suitable habitats (*p* > 0.7) [80]. The spatial distribution of potential habitats was visualized using ArcGIS 10.8, and the total area for each suitability class was calculated to quantify habitat availability across different suitability levels.

### 4.5. Analysis of Distribution Change

To analyze the dynamics of potential suitable habitats under climate change, two analytical approaches were employed. First, ASC result files from different time periods were converted into binary format using the “Quick Reclassify to Binary” function in SDMtools [81], with a threshold of 0.3. Presence probabilities exceeding 0.3 were assigned a value of 1, while values below 0.3 were assigned 0.

Next, the “Distribution Changes Between Binary SDMs” tool in SDMtools v2.5 was applied within ArcGIS v10.8 to compare binary distributions across different time periods and the current distribution. This allowed us to identify areas of stability, contraction, expansion, and absence under various scenarios. Furthermore, the centroids of suitable habitats for each period and scenario were calculated to trace their migration over time, providing insights into the spatial shifts of suitable habitats from the past to the future. The distances between centroids across consecutive time periods were also calculated to quantify the magnitude of these migrations.

## 5. Conclusions

Using the optimized MaxEnt model, we identified elevation, precipitation of the driest month (bio14), and isothermality (bio3) as the dominant environmental variables influencing the distribution of *C. smyrnioides*. Analysis of single-response curves for these variables revealed strong alignment with the species’ actual ecological conditions, further validating the model’s reliability. The current predictions indicate that the primary suitable habitats for *C. smyrnioides* are concentrated in Jiangsu, Anhui, Zhejiang, Hubei, and Hunan Provinces. Under future climate scenarios, new suitable habitats are projected to emerge in Shandong and Liaoning Provinces, particularly by the 2090s under the SSP5.85 scenario. Jiangsu Province is expected to remain the core region for high-suitability habitats, underscoring the species’ strong adaptability to local environmental conditions. In addition, our analysis revealed that *C. smyrnioides* tends to migrate to higher altitudes in response to climate change, likely as an adaptive strategy to mitigate the effects of rising temperatures. This altitudinal shift highlights the species’ resilience to warming climates and provides critical insights into its future distribution dynamics. Based on these findings, targeted conservation strategies are recommended to safeguard the species. Establishing small-scale protected areas in regions identified as highly suitable under current conditions is crucial to ensure in situ conservation. Artificial cultivation should be promoted in newly suitable areas, such as Shandong and Liaoning Provinces, to support population stability in response to climate change. For regions like Jiangxi and Hunan Provinces, where suitable habitats are projected to contract, germplasm conservation is essential to preserve genetic diversity and facilitate research into adaptive mechanisms. Furthermore, strengthening legal protections and enhancing public awareness are vital to mitigate anthropogenic pressures and support biodiversity conservation efforts. This study provides significant insights into the potential distribution and adaptive responses of *C. smyrnioides* to climate change. It offers a robust scientific foundation for developing effective conservation and cultivation strategies for this endangered species.

## Figures and Tables

**Table 1:** Comparison of the optimized parameter and default parameter.

Type	RM	FC	Mean AUC Ratio	Omission Rate at 5%	Delta AICc
Default	1	lqph	1.961	0	2048.245
Optimization	0.5	lq	1.973	0.059	0

**Table 2:** Suitable area of *C. smyrnioides* under various scenarios in China.

Climate Scenarios	Unsuitable Area (10^6^ Km)	Low-Suitability Area (10^4^ Km)	Moderately Suitable Area (10^4^ Km)	Highly Suitable Area (10^4^ Km)	Total Suitable Area (10^4^ Km)
Current	930.43	20.11	7.35	2.11	29.57
SSP1.26-2050s	922.23	12.88	7.87	17.03	37.78
SSP1.26-2070s	925.91	14.23	7.71	12.14	34.08
SSP1.26-2090s	929.68	16.28	8.25	5.79	30.32
SSP5.85-2050s	912.33	20.02	12.32	15.34	47.68
SSP5.85-2070s	893.09	38.17	11.09	17.66	66.92
SSP5.85-2090s	872.53	55.27	22.02	10.18	87.47

**Table 3:** Area changes of *C. smyrnioides* under various scenarios.

Climate Scenarios	Expansion (10^5^ Km)	Stable (10^5^ Km)	Contraction (10^4^ Km)
Current_SSP1.26-2050s	1.539	2.19	7.32
Current_SSP1.26-2070s	1.16	2.20	7.19
Current_SSP1.26-2090s	0.89	2.10	8.21
Current_SSP5.85-2050s	2.38	2.33	5.94
Current_SSP5.85-2070s	3.99	2.61	3.11
Current_SSP5.85-2090s	6.04	2.60	3.22

## Data Availability

Data are contained within the article and Supplementary Materials.

## Supplementary Materials

The following supporting information can be downloaded at https://www.mdpi.com/article/10.3390/plants14050743/s1, Table S1: Original occurrence records; Table S2: Filtered original occurrence records; Table S3: Environmental variables in bold were used in this study; Table S4: Percent contribution and permutation importance of variables; Figure S1: Heatmap of environmental variables.
