# Supplementary figures and images for: Prediction of Potential Distribution and Response of Changium smyrnioides to Climate Change Based on Optimized MaxEnt Model
Source: Plants (Basel). 2025 Feb 28;14(5):743. doi: 10.3390/plants14050743 (PMC11901656; doi:10.3390/plants14050743)

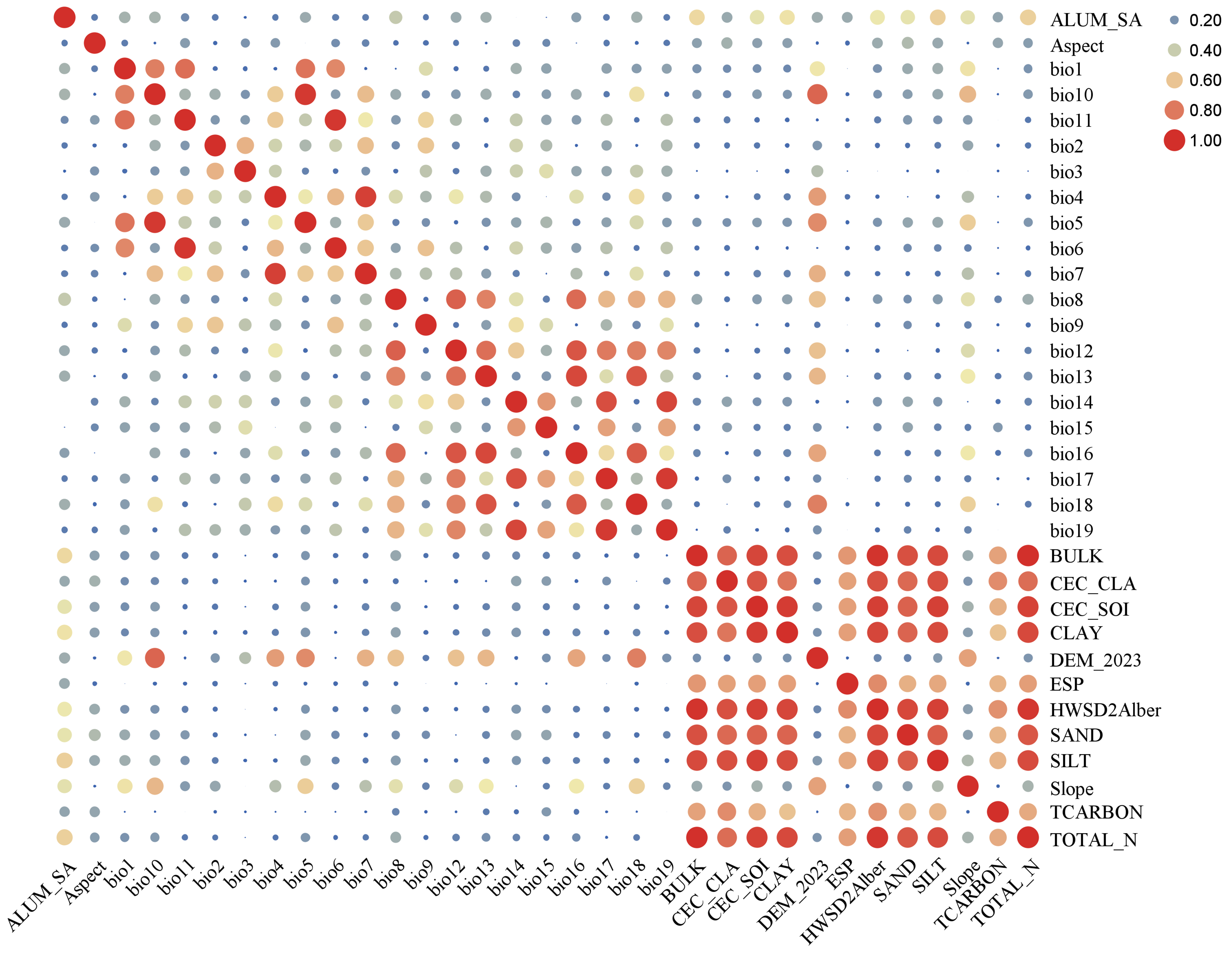

Supplement: Supplementary file 1 [file plants-14-00743-s001.zip › Figure S1.tif]
